# Exercise Intervention for Blood Pressure Reduction in Diabetic Patients: A Systematic Review and Meta-Analysis

**DOI:** 10.7759/cureus.104244

**Published:** 2026-02-25

**Authors:** Hakeem Adekunle, Olalekan Balogun

**Affiliations:** 1 Mathematics and Statistics (Biostatistics), Georgia State University, Atlanta, USA; 2 Microbiology, University of Ibadan, Ibadan, NGA

**Keywords:** blood pressure, diabetes, disease, exercise, health complication

## Abstract

Diabetes is a disease that occurs when the blood glucose is too high in the body. The disease has become a deadly epidemic due to its widespread and increasing prevalence. With the increasing prevalence of diabetic cases globally, and most especially in the United States of America, there is an urgent need to manage the disease by assessing the impact of exercise on blood pressure reduction in a diabetic patient. An electronic search was conducted in PubMed and Google Scholar for studies published between January 2005 and December 2023. The meta-analysis was performed without any restriction on patients’ gender, demographics, and ethnicity. The finding was reported following the Preferred Reporting Items for Systematic Reviews and Meta-Analyses guidelines (PRISMA) checklist. Six studies met the inclusion criteria for meta-analysis. Our result showed that the intervention group significantly caused a reduction in systolic blood pressure (SMD = -0.12 mmHg, 95% CI, -0.58 to 0.35; tau square = 0.3790; p < 0.0001) and diastolic blood pressure (SMD = -1.29 mmHg, 95% CI, -2.49 to -0.08; tau square = 2.9214; p < 0.0001) in patients with diabetes. The review demonstrated that regular exercise could positively impact patient health status and reduce the risk of death.

## Introduction and background

Diabetes is a disease that occurs when the blood glucose is too high in the body. The disease has become a deadly epidemic due to its widespread and increasing prevalence. Over 40 million people had diabetes in the United States, and about 12% worldwide [1]. Presently, it is estimated that about 115 million people also have pre-diabetes, a condition in which blood pressure (BP) levels are above normal, thus greatly increasing their risk for type 2 diabetes. Diabetes can lead to premature mortality and morbidity related to cardiovascular disease, blindness, amputation and kidney problems, and nerve disease.

Physical activity was defined as movement produced by the concentration of skeletal muscle that increases energy expenditure. Exercise, which is a subset of physical activity, can be taught with the intention of developing physical fitness, that is, strength and flexibility training. The intent of doing exercise is to know that several types of physical movement have a great effect on morbidity, physical fitness, and mortality in individuals with diabetes. Exercise is an important remedy to manage diabetes. According to research by Snowling et al. [2], it was reported that walking, jogging, or cycling, classified as aerobic exercise, reduced the absolute BP value by approximately 0.6%. According to the American College of Sports Medicine (ACSM), exercise causes an increase in glucose uptake into active muscle, balanced by hepatic glucose production with a greater reliance on carbohydrate to fuel muscular activity as intensity increases, thereby reducing BP. The maintenance of normal BP at rest and during exercise depends largely on the integration and coordination of the sympathetic nervous and endocrine systems [3].

The study by Cornelissen et al. [4] reported that either aerobic or resistance exercise is associated with BP lowering in individuals without diabetes. However, several meta-analysis studies have reported the effect of physical activities in the reduction of BP in patients with diabetes. There is an argument in the results of four meta-analyses that assessed the effect of cardiovascular diseases in patients with diabetes, whether exercise affects systolic BP (SBP) and diastolic BP (DBP) changes after different types of physical training [5-7]. The study did not address exercise as a single intervention and also did not address the publication bias and heterogeneity across the studies; as such, there is a need for a new systematic review and meta-analysis addressing the relationship between different BP and exercise in patients with diabetes. Therefore, the aim of this current systematic review and meta-analysis of a randomized controlled trial was to investigate the effect of BP on exercise (either aerobic, resistance, or combined exercise) in patients with diabetes.

## Review

Methodology

Inclusion and Exclusion Criteria

For this research, we include only randomized control design studies published from January 2005 to December 2023, written in the English language, and full-text studies on exercise/physical activities/training, and diabetes. We exclude studies written in languages other than English, and we exclude reviews, case studies, journal articles, and conference studies.

Data Source and Search Strategy

A systematic review and meta-analysis, in accordance with the Preferred Reporting Items for Systematic Reviews and Meta-Analyses guidelines (PRISMA), was performed [8]. PubMed and Google Scholar were searched for the publication period between January 2005 and December 2023. The scope of the literature search was based on the Population, Intervention, Comparison, and Outcome (PICO) format. The population (P) of interest was children and adult patients with diabetes; the intervention (I) was exercise; the control (C) was any other activity; the outcome (O) was a reduction in BP biomarkers.

The literature search was performed using the following specific keys: [“Physical activity” OR “Exercise” OR “Training”] AND [“Blood Pressure” OR “BP”] AND [“Diabetes” OR “type 2 diabetes’]. And the Mesh term: (((("Physical activity"[All Fields] OR "Exercise"[All Fields] OR "Training"[All Fields]) AND "Blood Pressure"[All Fields]) OR "BP"[All Fields]) AND "Diabetes"[All Fields]) OR ("diabetes mellitus, type 2"[MeSH Terms] OR "type 2 diabetes mellitus"[All Fields] OR "type 2 diabetes"[All Fields])

Quality Assessment

For a randomized control trial, we adopted the risk of bias assessment (ROB2) proposed by Sterne et al. [9]. Each study was assessed based on six criteria: Random sequence generation, allocation concealment, blinding of participants and personnel, Blinding of outcome assessment, incomplete outcome data, selective reporting, and other bias. For each of these criteria, we rate each study as either low (green color), unclear (unclear), or high (red).

Data Extraction

A pre-defined sheet was used to gather the following information from the included studies; study design, author first name, year of publication, total number of patients with diabetes, type of BP reported, the intervention and the control group, number patients in each group, summary statistics such as mean and standard of both SBP and DBP (Appendix 1 and Appendix 2). A PRISMA flowchart was subsequently generated for the final selection of studies to be included (see Results).

Data Evaluation and Analysis

A random-effects meta-analysis was performed using RevMan Version 5.4. The random-effects model was chosen because it was unknown whether there was a ‘true’ effect size underlying all studies, which would indicate the use of a fixed-effects meta-analysis; thus, we selected a more conservative approach. The chosen effect size is the standardized mean difference (SDM) with its associated 95% confidence intervals (CIs). The I² test was used to assess the magnitude of heterogeneity. A 5% CI was used, and the possibility of publication bias was assessed using the funnel plot.

Results

Study Selection

**Figure 1:**
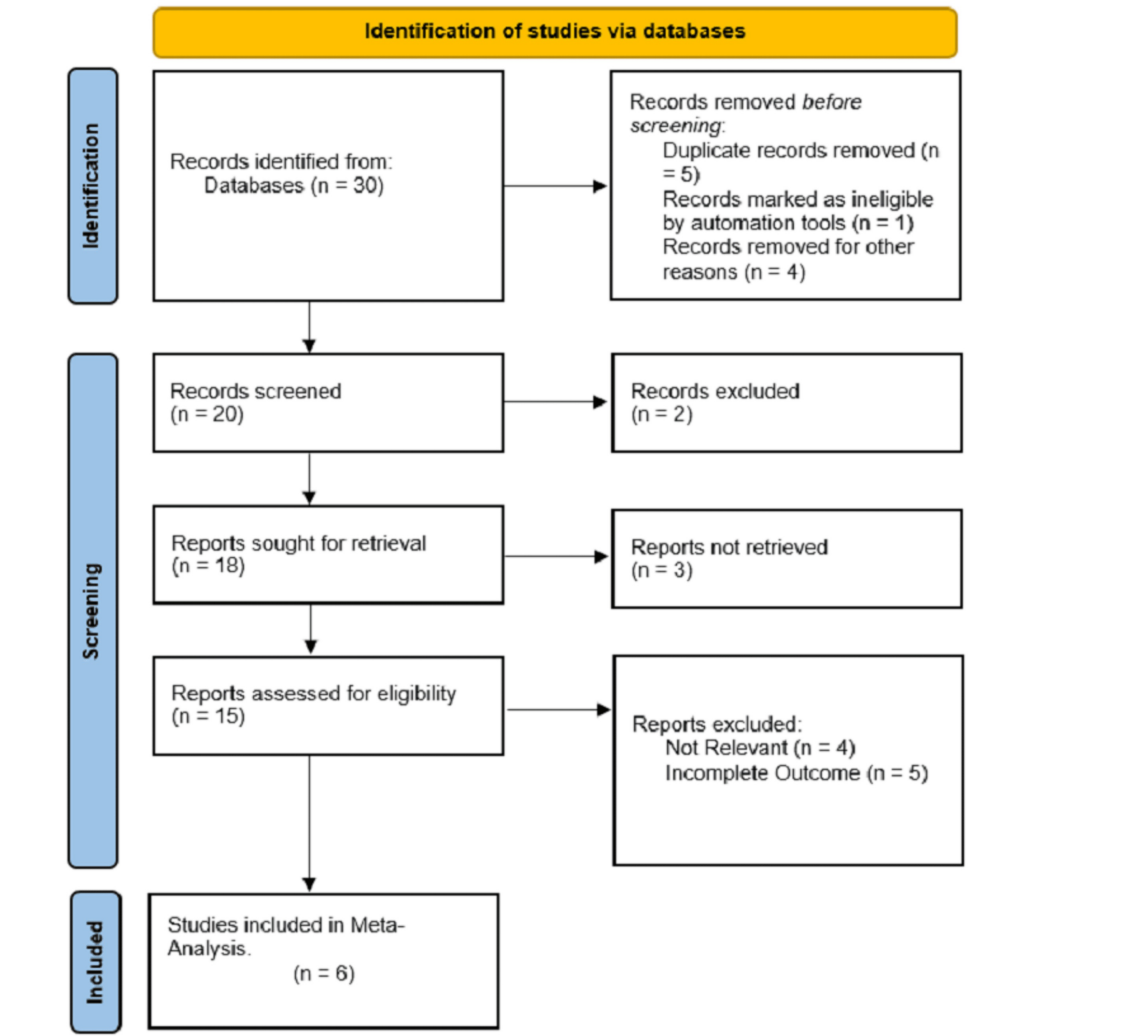
PRISMA (Preferred Reporting Items for Systematic Reviews and Meta-Analyses) flowchart for study inclusion

A total of 30 hits was generated from the database. After removing duplicates and irrelevant studies, six articles were included in the quantitative and qualitative analysis. The results were summarized in the PRISMA flow chart (Figure 1).

Characteristics of the Included Studies

A total of 1112 patients were included in the meta-analysis, with a minimum mean age of 53.4 years and a maximum of 61.8 years. The patients were randomized into either an exercise group (aerobic and resistance training) or a control group. The maximum reported follow-up period was 12 months, and the lowest was three months. The average duration of diabetes ranges from five years to 30 years (Table 1).

**Table 1:** Summary of the characteristics of the included studies

First Author	Study Design	Population Size	Age (M ± SD, range)	Gender M/F	Intervention	Control	Duration of Diabetes (years)	Medications	Follow-up
Paula et al. 2015 [10]	RCT	40	61.8±8.1	16/24	Exercise	Dietary recommendations	16.9±7.9	Aspirin	-
Sigal et al. 2007 [11]	RCT	251	53.5±0.0	160/91	Aerobic, resistance exercise	Dietary intervention	AT = 5.1 ± 3.5, RT = 6.1 ± 4.7, Control = 5.0 ± 4.5	Metformin, sulfonylurea, meglitinide, thiazolidinedione, glucosidase inhibitor	26 week
Balducci et al. 2010 [12]	RCT	606	≥ 60	-	Exercise	Standard care	-	Sulfonylureas, meglitinides, metformin, acarbose, thiazolidinediones	12 Months
Dobrosielski et al. 2012 [13]	RCT	140	56±6	81/59	Exercise	Dietary guidelines	30 ± 5	Metformin, sulfonylurea, meglitinide, thiazolidinedione	6 Months
Jorge et al. 2011 [14]	RCT	48	54.10±8.94	24/24	Aerobic, resistance exercise	Light stretching exercise	7.70±3.90	Sulfonylurea, metformin, DPP-4	12 weeks
Kadoglou et al. 2007 [15]	RCT	54	56.83±6.7	-	Exercise	Habitual activities	6.5±4.64	Sulfonylurea, metformin	16 weeks

Quality Assessment of the Included Studies

**Figure 2:**
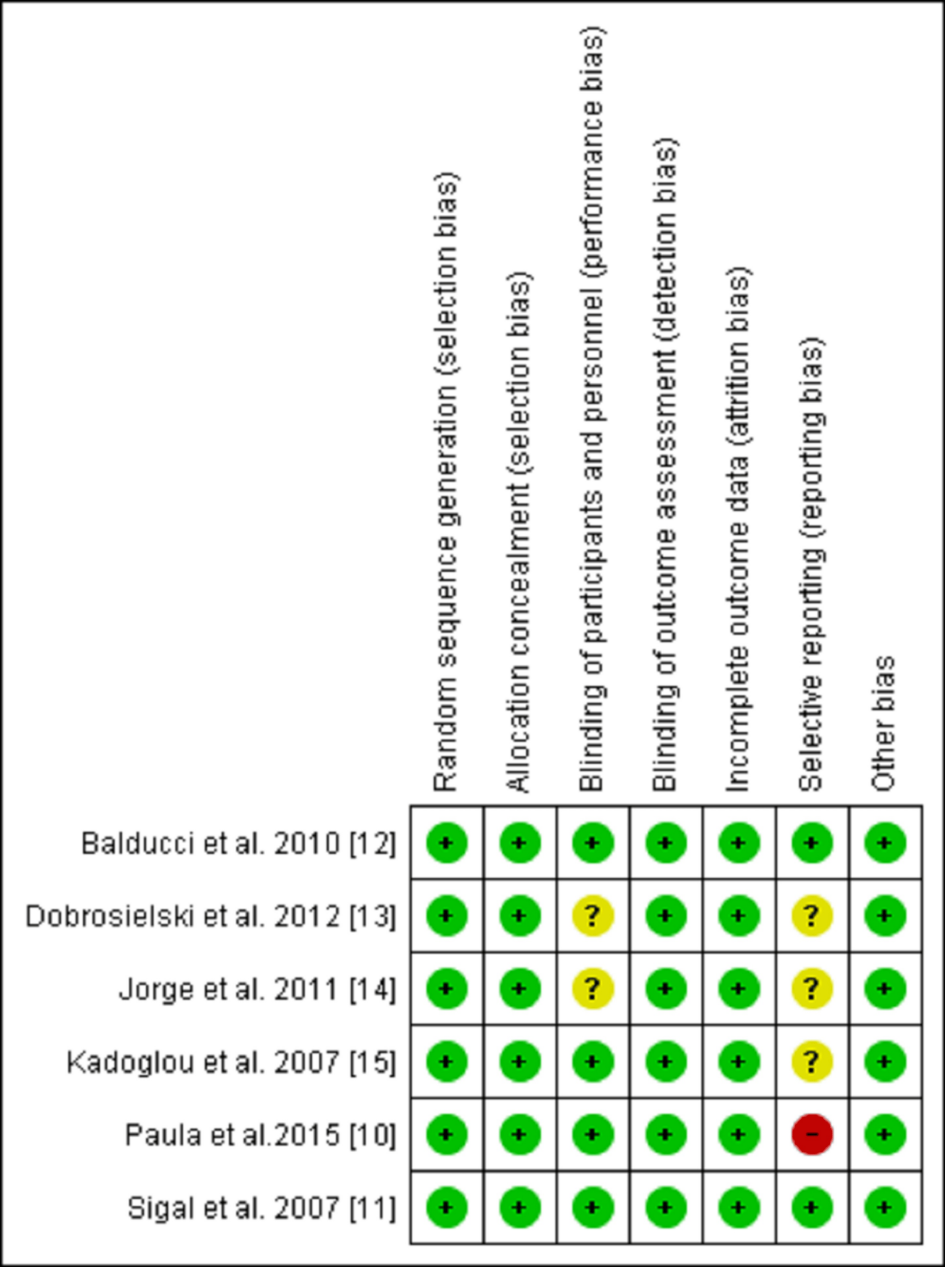
Summary of the risk of bias results using ROB 2. Green color (+) represents low risk, yellow color (question mark) represents an unclear risk, and red color represents a high risk.

Based on the results of the risk of bias assessment, two studies had a low risk across all six criteria (Sigal et al. 2007, Balducci et al. 2010). One study had a high risk of bias under the selective reporting bias and low risk of bias in other criteria (Dobrosielski et al. 2012), two studies had an unclear risk of bias under the blinding of participants and selective reporting bias and low risk of bias in other criteria (Dobrosielski et al. 2012, Jorge et al. 2011). The study by Kadoglou et al. (2007) reported an unclear risk of bias under the selective reporting bias and low risk in other criteria (Figure 2).

Meta-Analysis

**Figure 3:**
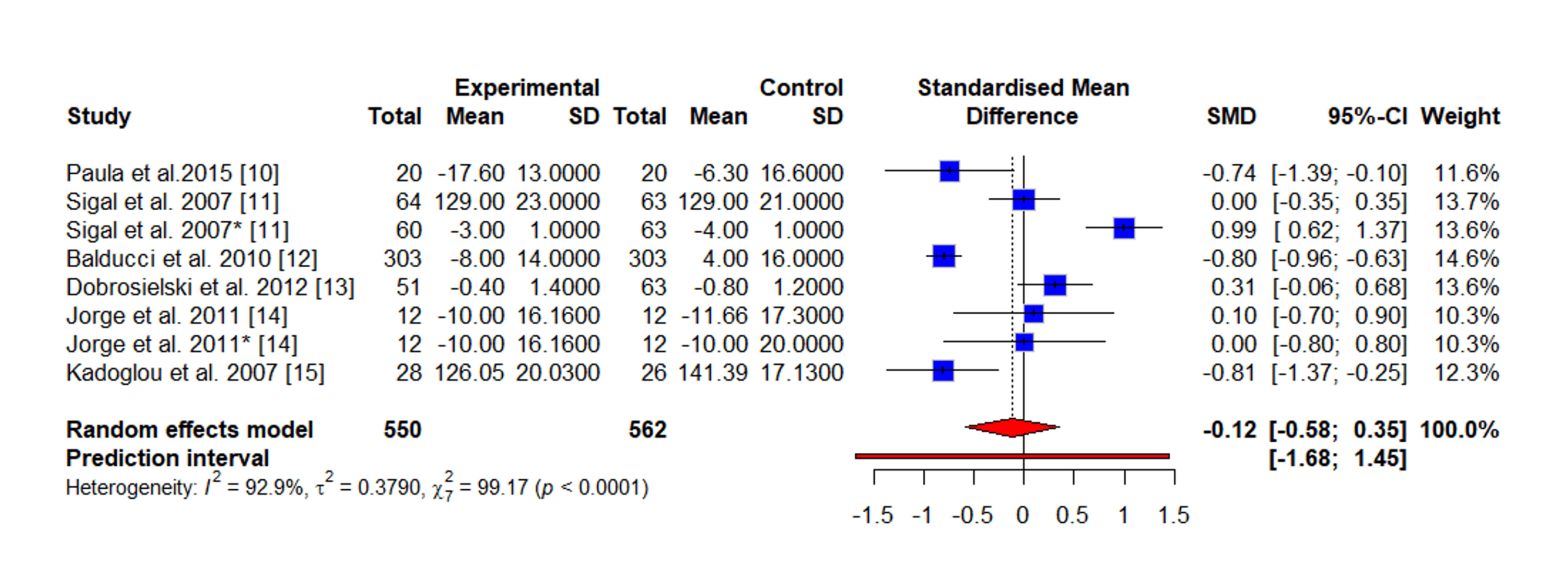
Forest plot of meta-analysis of the outcome (SBP) comparing the exercise training group (experimental) versus the control group The study by Sigal et al. and Jorge et al. reported two interventions (aerobic and resistance): The (*) represents the study with resistance exercise (i.e., Sigal et al. 2007* [11], Jorge et al. 2011* [14])

**Figure 4:**
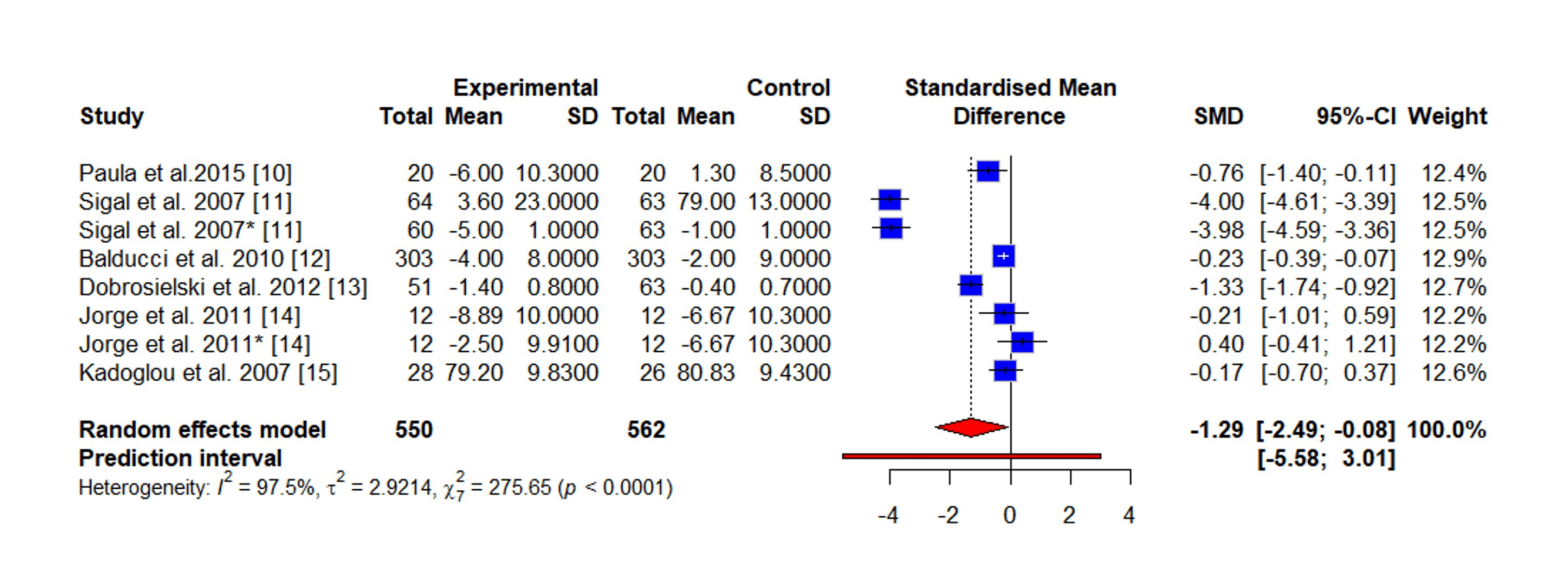
Forest plot of the meta-analysis of the outcome (DBP) comparing the exercise group and the control group The study by Sigal et al. and Jorge et al. reported two interventions (aerobic and resistance): The (*) represents the study with resistance exercise (i.e., Sigal et al. 2007* [11], Jorge et al. 2011* [14])

**Figure 5:**
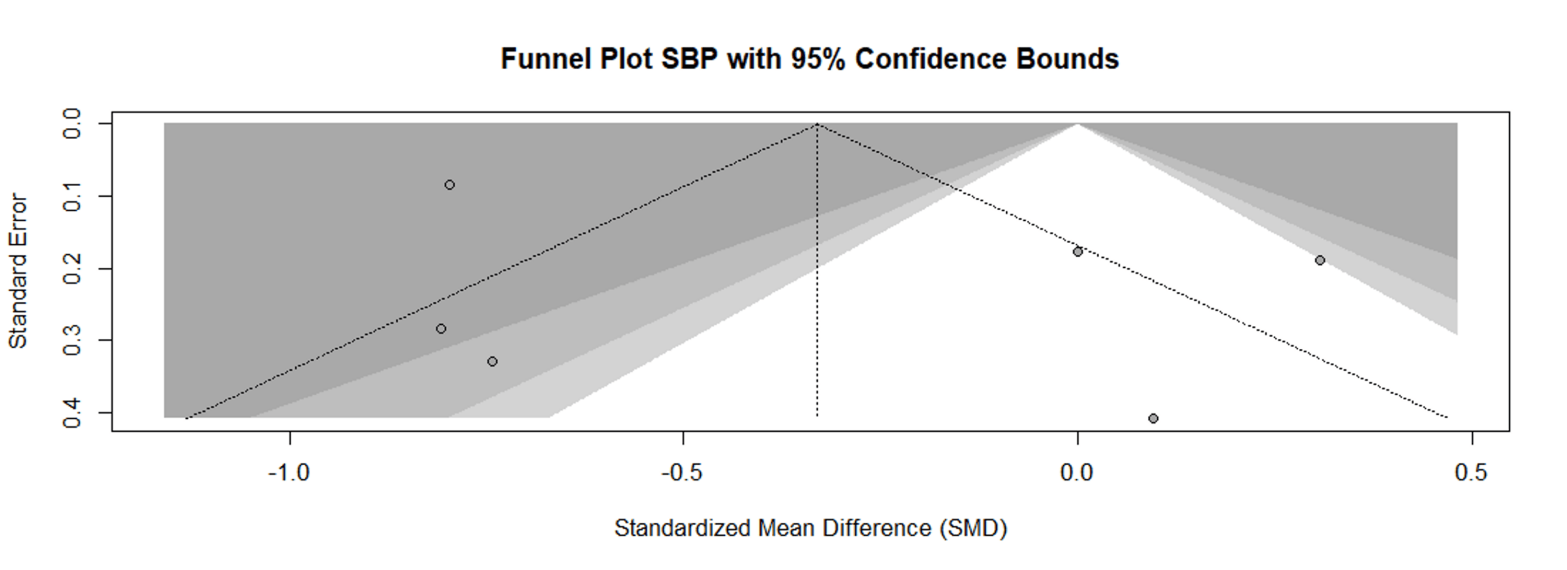
Funnel plot of the publication bias of studies included in the meta-analysis of the SBP SBP: Systolic blood pressure

**Figure 6:**
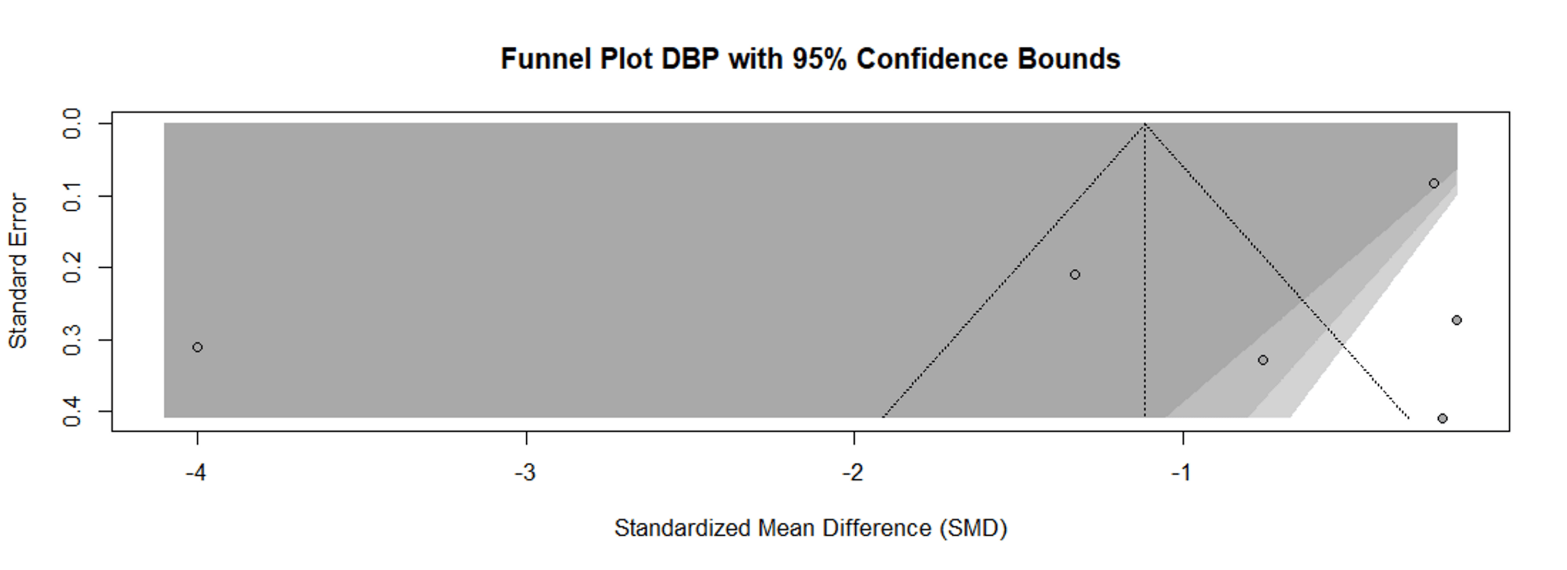
Funnel plot of the publication bias of studies included in the meta-analysis of the DBP DBP: Diastolic blood pressure

The pooled results of the meta-analysis of the outcome (SBP) demonstrated a clear beneficial effect of the intervention. The intervention group achieved a statistically significant reduction in SBP compared to the control group (SMD = -0.12 mmHg, 95% CI, -0.58 to 0.35; tau square = 0.3790; p < 0.0001), as detailed in Figure 3. Similarly, for the meta-analysis of the outcome (DBP), the intervention group showed a significant overall reduction in DBP compared to the control (SMD = -1.29 mmHg, 95% CI, -2.49 to -0.08; tau square = 2.9214; p < 0.0001), as presented in Figure 4. A high degree of statistical heterogeneity was evident across the included studies for both SBP (I^2^ = 92.9%, p < 0.001) and DBP (I^2^ = 97.5%, p < 0.0001). This substantial heterogeneity may be attributable to differences in study characteristics, such as variations in sample size, diagnostic criteria for diabetes, and intervention methodology among the primary reports. Furthermore, the funnel plot of the study included in the meta-analysis of SBP (Figure 5) and DBP (Figure 6) indicated visual evidence of some distinct outlier studies.

Discussion 

To the best of our knowledge, we have shown that there is a significant association between exercise and patients with diabetes by reducing the level of both the SBP and DBP. Our results are similar to a meta-analysis reported by Figueira et al. [16], who reported a meta-analysis on the effect of aerobic exercise (AER), resistance exercise (RES), and high-intensity combined training on SBP and DBP in patients with type 2 diabetes. He found a significant association between structural exercise and patients with type 2 diabetes, with a reduction of SBP and DBP. He also found that AER and RES were not associated with BP reduction. Our study reported a high and significant heterogeneity value, which is true because there are clearly differences among the included studies, which may be a result of differences in country of publication, method of analysis, and type of physical training used in each study. This is not consistent with the study reported by Pan et al. [17] that found a low heterogeneity value of 55% in a study of resistance training. This implies that this value was not explained by clinical factors such as baseline BP, training intensity, and variables of training amount [18].

It is important to point out some of the limitations encountered in this study while interpreting the results. The first limitation was the small number of included studies, which may affect the conclusion of the study; a small number of studies in a meta-analysis leads to a smaller total number of patients. The second limitation was a lack of access to some of the studies due to the scarcity of studies on the present topic. Future researchers may utilize this opportunity to include more studies and perform analysis on the effect of different types of exercises on the two BPs in patients with type 2 diabetes.

## Conclusions

In summary, this systematic review and meta-analysis provide strong evidence that exercise intervention is an effective strategy for BP reduction in patients with diabetes. The pooled results demonstrate a statistically significant and clinically relevant reduction in both SBP and DBP. Specifically, rigorous exercise was associated with an average reduction of -0.12 mmHg in SBP and -1.29 mmHg in DBP. These findings support the inclusion of structured exercise programs as a key component of non-pharmacological management for BP control in the diabetic population.

## Appendices

Appendix 1

**Table 2:** Meta-data for SBP in mmHg The study by Jorge et al. 2011 [14] included a population size of 48, and only 12 patients were randomized to the intervention group and 12 to the control group. The study by Kadoglou et al. 2007 [15] included 140 patients, but 51 were randomized to the intervention group and 53 to the control group.

First Author	Study Design	Study Population	Intervention (n)	Mean Change from Baseline to Final	Standard Deviation	Control(n)	Mean Change from Baseline to Final	Standard Deviation
Paula et al. 2015 [10]	RCT	40	20	160.9	16.2	20	163.6	19
Sigal et al. 2007 [11]	RCT	251	64	129	23	63	129	21
Balducci et al. 2010 [12]	RCT	29	20	6.5	3	9	141	18
Dobrosielski et al. 2012 [13]	RCT	140	21	6.3	25	10	152	19
Jorge et al. 2011 [14]	RCT	48	12	10.5	13	12	135	15
Kadoglou et al. 2007 [15]	RCT	140	51	-5.3	2.5	53	4.5	2.2

Appendix 2

**Table 3:** Meta-data for DBP in mmHg The study by Jorge et al. 2011 [14] reported a population size of 48, but only 12 patients received resistance exercise, 12 received aerobic exercise, and 12 were assigned to the control group; the remaining 12 were not included at the endpoint. The study by Kadoglou et al. 2007 [15] included 140 patients, but only 51 received the intervention, and 53 received the control training.

First Author	Study Design	Study Population	Intervention (n)	Mean Change from Baseline to Final	Standard Deviation	Control	Mean Change from Baseline to Final	Standard Deviation
Paula et al. 2015 [10]	RCT	40	20	85.3	12	20	86.3	90
Sigal et al. 2007 [11]	RCT	251	64	3.6	23	63	79	13
Balducci et al. 2010 [12]	RCT	29	20	15.6	3	9	84	10
Dobrosielski et al. 2012 [13]	RCT	140	21	45.1	25	10	85	10
Jorge et al. 2011 [14]	RCT	48	12	36.9	13	12	86	8
Kadoglou et al. 2007 [15]	RCT	140	51	-10.5	2.5	53	-1.5	0.2
